# Regulation of *per* and *cry* Genes Reveals a Central Role for the D-Box Enhancer in Light-Dependent Gene Expression

**DOI:** 10.1371/journal.pone.0051278

**Published:** 2012-12-06

**Authors:** Philipp Mracek, Cristina Santoriello, M. Laura Idda, Cristina Pagano, Zohar Ben-Moshe, Yoav Gothilf, Daniela Vallone, Nicholas S. Foulkes

**Affiliations:** 1 Institute of Toxicology and Genetics, Karlsruhe Institute of Technology, Eggenstein-Leopoldshafen, Germany; 2 Department of Stem Cell and Regenerative Biology, Harvard University, Cambridge, Massachusetts, United States of America; 3 Department of Neurobiology, George S. Wise Faculty of Life Sciences, Tel-Aviv University, Tel-Aviv, Israel; Vanderbilt University, United States of America

## Abstract

Light serves as a key environmental signal for synchronizing the circadian clock with the day night cycle. The zebrafish represents an attractive model for exploring how light influences the vertebrate clock mechanism. Direct illumination of most fish tissues and cell lines induces expression of a broad range of genes including DNA repair, stress response and key clock genes. We have previously identified D- and E-box elements within the promoter of the zebrafish *per2* gene that together direct light-induced gene expression. However, is the combined regulation by E- and D-boxes a general feature for all light-induced gene expression? We have tackled this question by examining the regulation of additional light-inducible genes. Our results demonstrate that with the exception of *per2*, all other genes tested are not induced by light upon blocking of *de novo* protein synthesis. We reveal that a single D-box serves as the principal light responsive element within the *cry1a* promoter. Furthermore, upon inhibition of protein synthesis D-box mediated gene expression is abolished while the E-box confers light driven activation as observed in the *per2* gene. Given the existence of different photoreceptors in fish cells, our results implicate the D-box enhancer as a general convergence point for light driven signaling.

## Introduction

The circadian clock is a highly conserved, physiological timing mechanism that allows organisms to anticipate and adapt to daily environmental changes [1]. At the core of the vertebrate circadian clock mechanism are interlocking transcription translation feedback loops that are composed of activator and repressor clock proteins [2]. The main loop consists of the positive elements CLOCK and BMAL, which form heterodimers that activate the transcription of the negative elements, *period* (*per*) and *cryptochrome* (*cry*). As the levels of PER and CRY rise, they enter the nucleus as heterodimers and repress their own transcription by inhibiting the action of the CLOCK:BMAL complex, thus closing the feedback loop [3]. This mechanism also involves additional stabilizing loops [4], [5] as well as complex posttranslational regulation. This additional regulation confers robustness and ensures that the mechanism requires *circa* 24 hours to complete one cycle [6]. Given that this is not a precise 24 hours clock, it is vital that its phase is reset regularly by signals that reliably indicate the time of day, to ensure its synchronization with the natural day-night cycle. The most commonly employed environmental signal or so-called “zeitgeber” is light although others include temperature and food availability [7], [8]. Given the importance of light as an environmental timing cue, most organisms have evolved dedicated photoreceptors and associated signalling pathways that relay this lighting information to the core clock machinery.

The zebrafish has been established as an attractive vertebrate model for studying key aspects of the light signalling pathway and its impact on the circadian clock [9], [10]. As in other vertebrates, most zebrafish tissues contain independent circadian clocks (so-called peripheral clocks) [11], [12]. While in mammals, light entrainment of peripheral clocks occurs indirectly via the retina and the central clock of the suprachiasmatic nucleus [13], in zebrafish the peripheral clocks are directly entrained by exposure to light [14], [15]. In zebrafish organs, tissues and cultured cells, exposure to light directly activates the transcription of two clock genes *per2* and *cry1a* that is predicted to lead to the entrainment of the circadian clock [16], [17], [18]. More recent transcriptome profiling studies in zebrafish have demonstrated that numerous other genes with different cellular functions including transcriptional control, stress response and DNA repair are also directly regulated by light [19], [20]. Thus, a key question is how does light exposure trigger changes in transcription?

We have previously shown that functional E- and D-box enhancer elements are both necessary and sufficient for light-regulated *per2* gene expression [21]. Moreover, we have revealed an enrichment of these enhancers in the promoters of light-induced genes [19]. However, little is known about the relative contribution of these two promoter elements to light regulated gene expression. Furthermore, do all light regulated genes share a common regulatory mechanism based on D- and E-boxes?

Here we have examined the regulation of gene expression in additional light inducible genes. We demonstrate that with the exception of *per2*, all other genes tested are not induced by light upon blocking of *de novo* protein synthesis. In the case of *per2*, inhibition of translation causes only a delay in induction following light exposure. In order to identify light responsive promoter elements in genes that rely upon protein synthesis, we performed a systematic functional analysis of the *cry1a* promoter in transfected zebrafish PAC-2 cells. We demonstrate that a single D-box directs light-induced expression of this clock gene. Furthermore, we show that light driven gene expression mediated by the D-box enhancer relies upon *de novo* protein synthesis. Interestingly, expression directed by E-box enhancer elements such as that in the *per2* promoter is increased in a light dependent fashion upon inhibition of protein synthesis. Thus our results support the notion that the D-box serves as the primary light responsive promoter element in zebrafish cells although other enhancers such as the E-box may modulate its function in a promoter specific fashion. Furthermore, given the presence of multiple photoreceptors in fish [18], [22], [23], the D-box promoter element would seem to serve as a general convergence point for light driven signaling.

## Results

### Differential Effect of Cycloheximide on Light-induced Gene Expression

**Figure 1:**
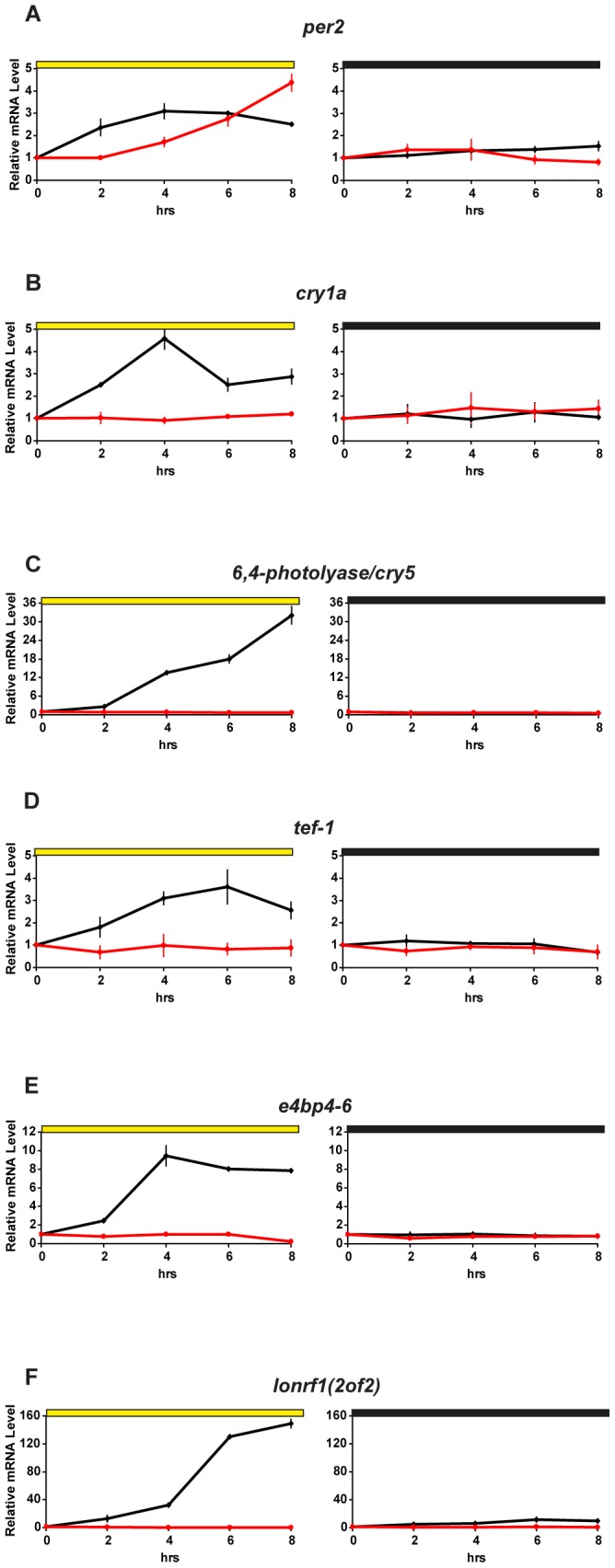
Differential effect of cycloheximide on light-induced gene expression. (A–F) Quantitative RT-PCR analysis (qRT-PCR) of light inducible genes in PAC-2 cells in the presence (red traces) or absence (black traces) of cycloheximide (CHX) during 8 hours of light exposure (left panels) or constant darkness conditions (right panels). Cells were maintained for 3 days in DD prior to the experiment. 1 h before sampling, cells were treated with CHX (10 µg/ml). Each gene is indicated above its respective panels. Yellow and black bars above each panel indicate the light and dark periods, respectively. Relative mRNA levels are plotted on the y-axes and were set arbitrarily as 1 at time-point 0 hrs for each gene. Endogenous *β-actin* mRNA levels were not influenced by light or cycloheximide treatment and so these were used to normalize the expression of each gene (see Figure S1 B). Time (hrs) is plotted on the x-axes. In each panel, points are plotted as the means of three independent experiments +/− SD. All statistical analyses (t-test and two-way ANOVA) are presented in Table S2. The blocking of protein synthesis by cycloheximide treatment of PAC-2 cells was confirmed in Figure S1 A.

In addition to the clock genes *per2* and *cry1a*, many other genes are acutely induced upon light exposure in zebrafish [19], [20], [24]. Do all these genes share a common light responsive regulatory mechanism? In support of the involvement of multiple pathways, previous studies have indicated differences between light induced *per2* and *cry1a* expression in terms of their requirement for *de novo* protein synthesis [25]. In order to explore this property in more detail, we chose to test the effect of cycloheximide treatment on light induced expression of a broader set of genes. We thus incubated zebrafish PAC-2 cells with cycloheximide (CHX) during light exposure and then sampled RNA at different time points for subsequent qRT-PCR analysis (Figure 1, Figure S1 and Table S1). As expected, without CHX-treatment, all genes were significantly up-regulated by light compared with constant dark controls (Figure 1, black traces and Table S2 A). Interestingly, the light-induced expression of *cry1a*, *6,4-photolyase/cry5*, *tef-1*, *e4bp4-6* and *lonrf1 (2 of 2)* was strongly inhibited upon treatment with CHX (Figure 1B–F, left panels, red traces and Table S2 A and B). However, light-induced *per2* expression still persisted after CHX treatment (Figure 1A, left panel, red trace and Table S2 A and B), although, with a significant delay compared to control non-CHX treated cells (p<0.0001, two-way ANOVA). Comparable results were obtained using two alternative protein synthesis inhibitors (puromycin and anisomycin, Figure S2). Thus, the majority of genes tested require *de novo* protein synthesis for light induced expression with *per2* being an exception.

### Role of AP-1 Enhancer Elements in Light-induced Expression of *cry1a*

**Figure 2:**
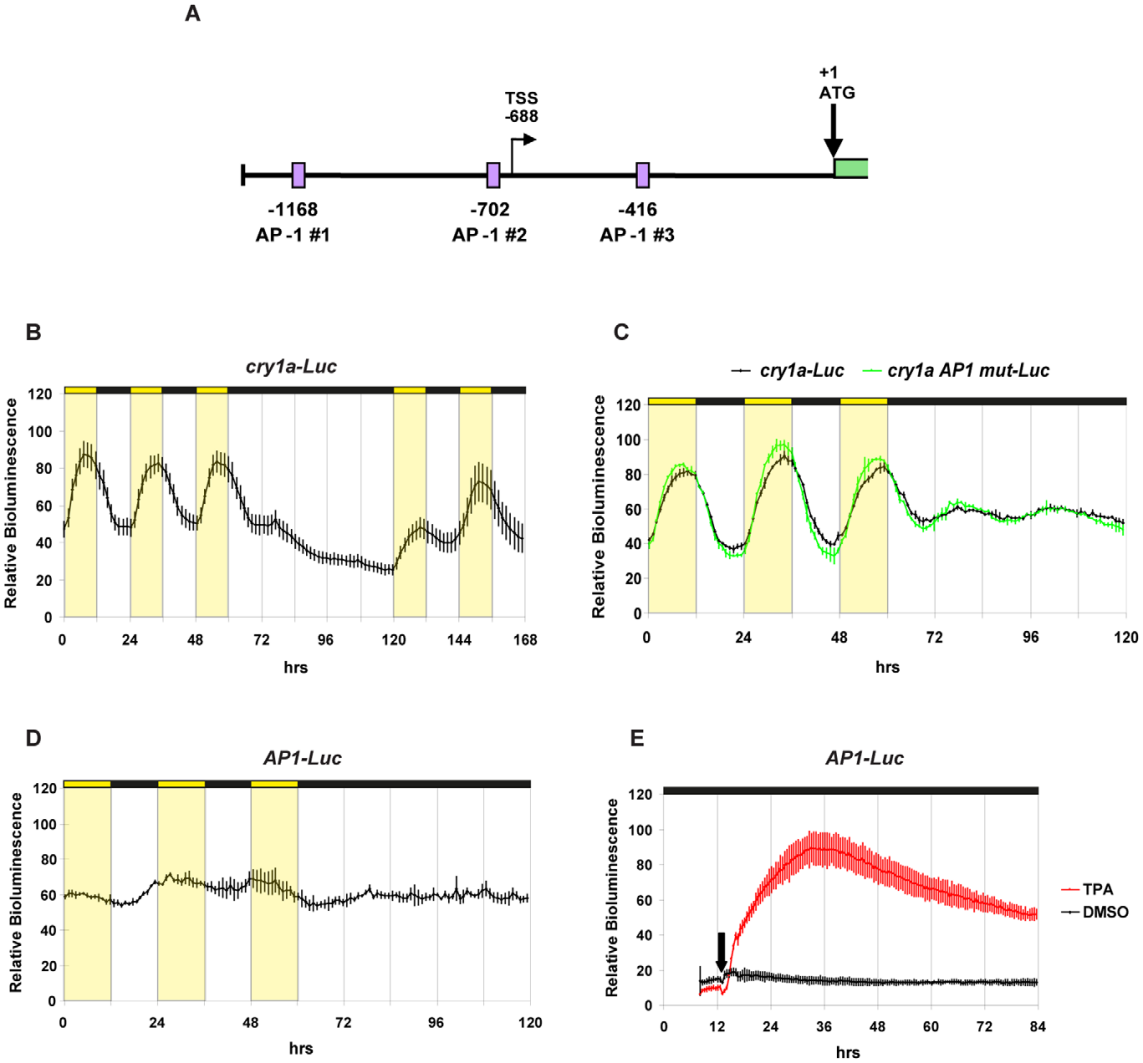
Role of AP-1 enhancer elements in light-induced expression of *cry1a*. (A) Schematic representation of the 1.3 kb *cry1a* promoter. The 53 bp exon 1 is indicated by a green rectangle. The transcription start site (TSS) at position −688 bp and the ATG at position +1 bp are indicated. Violet rectangles denote the three AP-1 sites (AP-1 #1 at position −1168 bp, AP-1 #2 at position −702 bp and AP-1 #3 at position −416 bp). (B – E) Representative real time bioluminescence assays of PAC-2 cells transfected with the following constructs (B) *cry1a-Luc*. (C) *cry1a-Luc* (black trace) and *cry1a AP1 mut -Luc* (green trace). (D) *AP1-Luc*. (E) *AP1-Luc* in the presence (red trace) or absence (black trace) of 50 ng/ml of the phorbol ester TPA. The black arrow indicates the time of TPA or DMSO-control addition. In each panel relative bioluminescence is plotted on the y-axis and time (hrs) on the x-axis. Each time-point represents the mean of at least four independently transfected wells +/− SD from a single experiment. Each experiment was performed a minimum of three times. Yellow and black bars above each panel represent the light and dark periods, respectively.

To date, the only systematic, functional promoter analysis of a light regulated zebrafish gene has revealed that functional E- and D-boxes are both necessary and sufficient for the light regulated expression of the *per2* gene [21]. Does a distinct mechanism operate in other light induced genes that rely on protein synthesis, such as *cry1a*? Interestingly, previous studies [25] have implicated two AP-1 sites as important transcriptional regulatory elements in the zebrafish *cry1a* gene in response to light. Besides the two AP-1 sites described by Hirayama et al. (AP-1 #1 at position −1168 bp and AP-1 #2 at position −702 bp, relative to the ATG), we have also identified a third potential AP-1 site located at position −416 bp (5′-TGAGTTA-3′) which we have termed AP-1 #3 (Figure 2A). To directly test the functionality of these elements, we initially cloned a genomic DNA fragment of 1.3 kb lying upstream of the *cry1a* gene (including 1.25 kb of 5′ flanking genomic DNA and 53 bp of exon 1) in a luciferase reporter vector (Figure 2A, *cry1a-Luc*). Consistent with this fragment representing the *cry1a* promoter, it encompasses the principal start site of transcription of the *cry1a* gene (at position −688 bp) as defined by 5′ RACE PCR [25]. Furthermore, a real-time bioluminescence assay of zebrafish PAC-2 cells stably transfected with the *cry1a-Luc* construct revealed robust rhythms of luciferase activity under light-dark (LD) cycle conditions (Figure 2 B, Table S3). An increase in expression was observed during the beginning of the light phase and a decrease just preceding the onset of the dark phase.

To test the relevance of the three AP-1 sites for the *cry1a* light-responsiveness we mutated all three sites present in *cry1a-Luc* generating *cry1a AP1 mut-Luc* and then performed a real-time bioluminescence assay in PAC-2 cells (Figure 2C and Table S4). The cells were exposed to LD cycles and then transferred to constant darkness (DD) conditions. Surprisingly, our data revealed no difference in the light inducible expression pattern of *cry1a AP1 mut-Luc* compared with the control wild-type construct *cry1a-Luc* (Table S3). Thus, our results do not support a significant role for the AP-1 enhancer in the light-driven regulation of the *cry1a* promoter. To confirm the lack of light responsiveness of the AP-1 enhancer element, we generated a heterologous AP-1 reporter construct. The canonical AP-1 enhancer sequence (5′-TGACTCA-3′), located within the *cry1a* promoter at position −1168 bp (AP-1 site #1), was multimerized and inserted into a minimal promoter - driven luciferase reporter generating the *AP1-Luc* construct. In a real-time bioluminescence assay, the *AP1-Luc* construct showed no light-driven changes in expression (Figure 2D, Table S3). In contrast, the same construct was strongly activated upon phorbol ester 12-O-tetradecanoylphorbol-13-acetate (TPA) treatment as a control for AP-1 function [26] (Figure 2E). Together, these data clearly demonstrate that the three AP-1 enhancer elements investigated do not mediate light regulated gene expression in zebrafish PAC-2 cells.

### Identification of a Light-responsive Region within the *cry1a* Promoter

**Figure 3:**
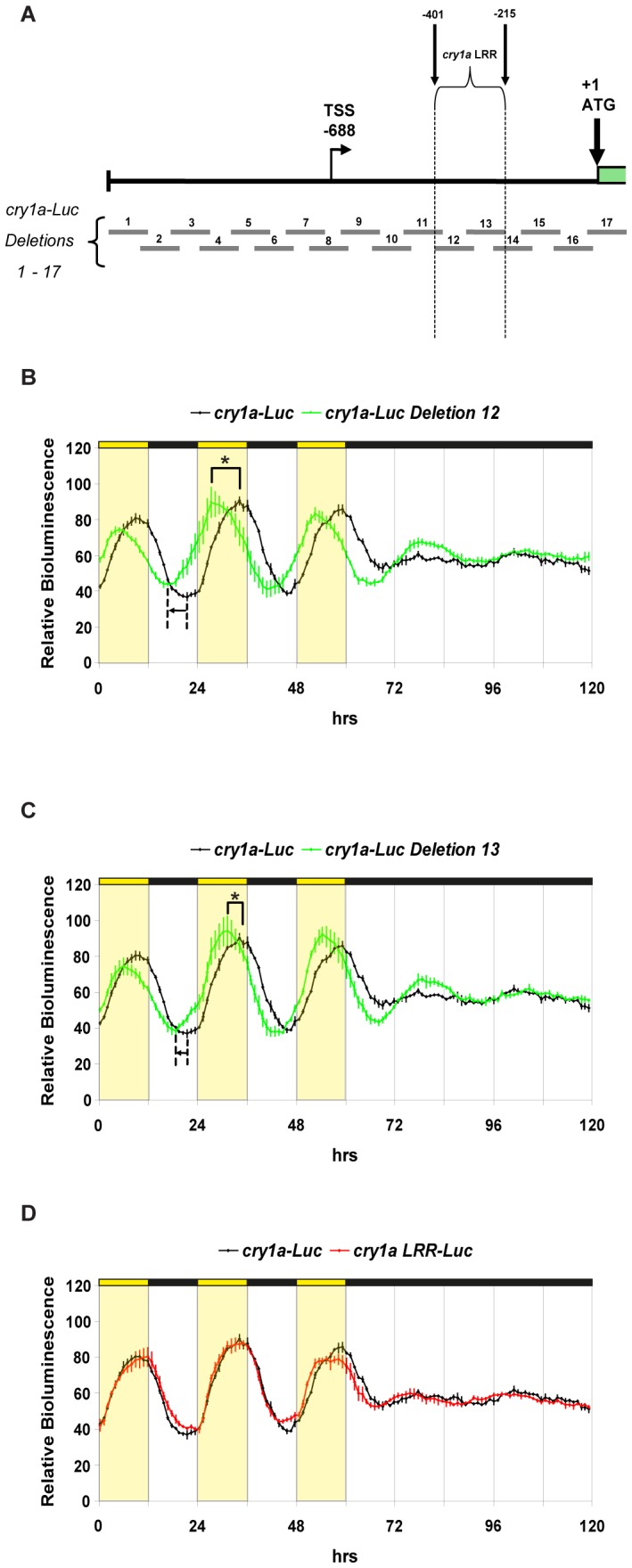
Identification of the light-responsive region within the *cry1a* promoter. (A) Schematic representation of *cry1a-Luc* and deletion constructs 1 to 17 (grey bars) (see also Table S5). The 186 bp *cry1a* light responsive region (*cry1a* LRR) is indicated by dotted lines and black arrows (region between −401 bp and −215 bp). (B–C) Representative real time bioluminescence assays from PAC-2 cells transfected with *cry1a-Luc* (black trace) and *cry1a-Luc Deletion 12* or *cry1a-Luc Deletion 13* (green traces) (Figure 3B and C). In both *cry1a-Luc Deletions 12* and *13*, the phase of rhythmic expression is significantly shifted (for both p<0.0001, t-test). In deletions 12 and 13, the increase in luciferase activity that anticipates the onset of the light phase is indicated by a horizontal black arrow. (D) Representative real time bioluminescence assay from PAC-2 cells transfected with *cry1a-Luc* (black trace) and *cry1a LRR-Luc* (red trace). In each panel relative bioluminescence is plotted on the y-axis and time (hrs) on the x-axis. Each time-point represents the mean of at least four independently transfected wells +/− SD from a single experiment. Each experiment was performed a minimum of three times. Yellow and black bars above each panel represent the light and dark periods, respectively. Statistically significant differences are indicated by an asterisk (*) and are reported in Table S3.

In order to identify which enhancer elements are crucial for the *cry1a* light induction, we prepared a series of seventeen partially overlapping deletions based on the *cry1a-Luc* wild-type construct (Figure 3A and Table S5). These deletion constructs were then transiently transfected in PAC-2 cells and tested for light-regulated expression (Figure 3B–C and Figure S3). In both deletions 12 and 13, the characteristic acute increase in expression observed immediately following “lights on” in the *cry1a-Luc* construct was absent. However, under LD cycles, both constructs do retain rhythmic expression (Figure 3B–C and Table S3), with an increase in luciferase activity anticipating the onset of the light phase, a feature characteristic of a circadian clock regulated promoter. This results in a shift in the phase of rhythmic expression relative to the full *cry1a-Luc* promoter (p<0.0001, t-test) and reveals a switch from light driven to clock regulated rhythmicity in these two deletion constructs. The remaining deletions did not exhibit any significant differences in terms of either light-induced expression or the phase of rhythmic expression with respect to the wild-type *cry1a-Luc* reporter (Figure S3 and Table S3). Interestingly, deletion of the transcription start site (TSS) failed to influence the basal levels of expression (see deletion 8 in Figure S3). This potentially points to the existence of additional alternative transcription start sites. Together, these results point to a region of 186 bp (−401 bp to −215 bp) containing the elements that are necessary for light-responsiveness of the zebrafish *cry1a* gene which we have termed the *cry1a* “light responsive region” (*cry1a* LRR). Furthermore, given the persistence of rhythmic expression even upon deletion of the LRR region, we predict the existence of a functional clock-regulated enhancer element lying outside of this region.

In order to test if the *cry1a* LRR is sufficient to drive light dependent rhythmic expression similar to the *cry1a-Luc* promoter construct, the LRR sequence was cloned into a luciferase reporter vector (*cry1a LRR-Luc*). This construct showed a comparable light driven expression pattern to the *cry1a-Luc* promoter construct (Figure 3D and Table S3). Thus, the LRR is sufficient to direct light-driven rhythmic gene expression.

### A Single D-box is Necessary and Sufficient for the Light Response of the *cry1a* Gene

**Figure 4:**
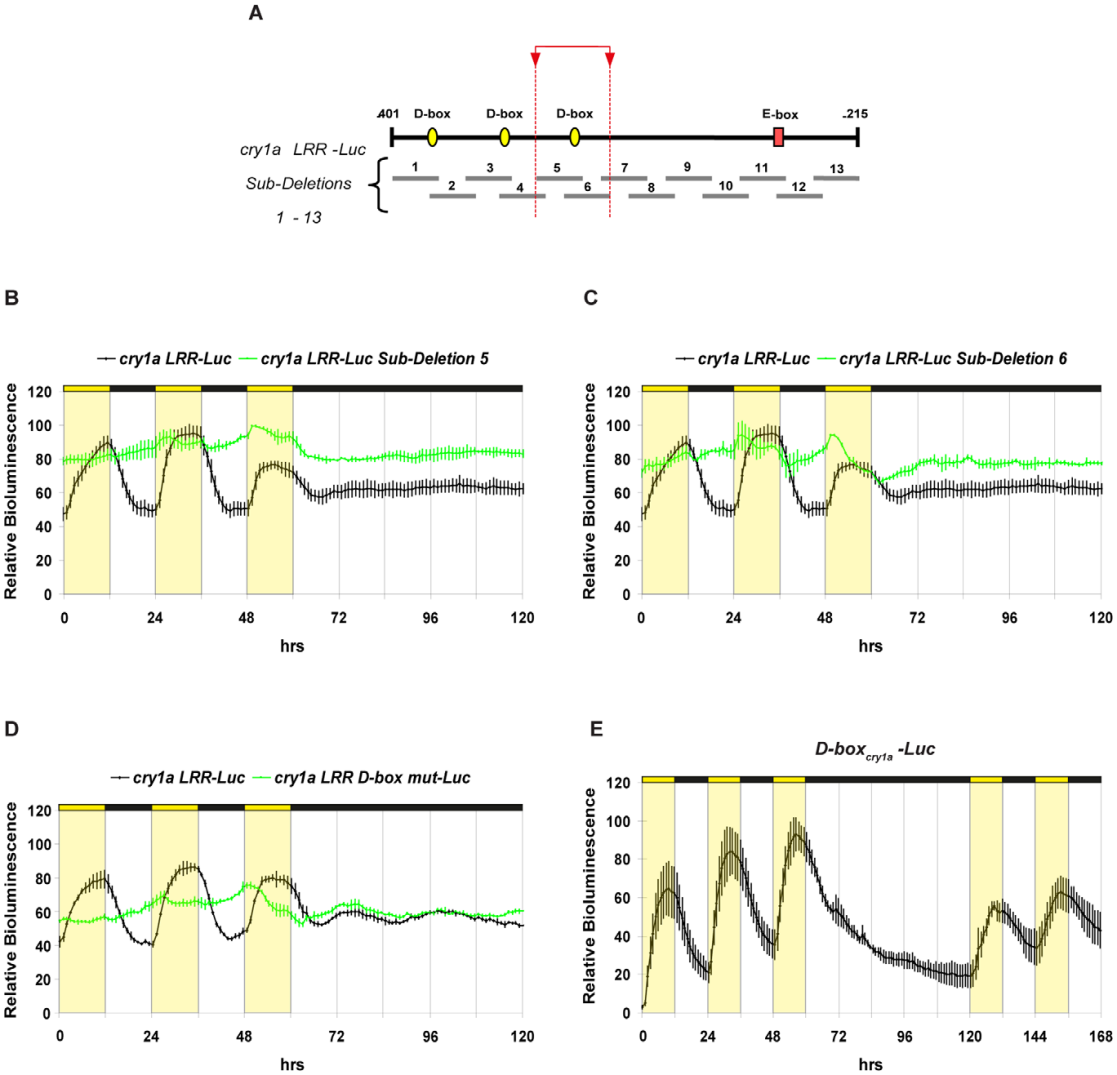
A single functional D-box is necessary and sufficient for the light response of the *cry1a* gene. (A) Schematic representation of *cry1a LRR- Luc* and sub-deletion constructs 1 to 13 (dark grey bars). The red rectangle denotes the putative E-box while the three yellow ellipses represent the putative D-boxes. The region delimited by *cry1a LRR-Luc Sub-Deletions 5* and *6* is indicated by red arrowheads and red dotted lines. This region includes the light responsive D-box. (B–E) Representative real time bioluminescence assays from transfected PAC-2 cells. The identity of the transfected constructs and their colour codes are indicated above each panel. In each panel relative bioluminescence is plotted on the y-axis and time (hrs) on the x-axis. Each time-point represents the mean of at least four independently transfected wells +/− SD from a single experiment. Each experiment was performed a minimum of three times. Yellow and black bars above each panel represent the light and dark periods, respectively. Statistically significant differences are reported in Table S3.

Interestingly, similar to the situation in the *per2* promoter [21], the *cry1a* LRR contains putative D-box and E-box enhancer sequences (Figure 4A). In order to determine whether these enhancer elements are responsible for the *cry1a* LRR light-induction we generated and tested a new series of thirteen partially overlapping sub-deletions within the *cry1a LRR-Luc* construct (Figure 4A and Table S5). Two sub-deletions, sub-deletion 5 and sub-deletion 6, showed a complete disruption of the characteristic light inducible expression pattern of the *cry1a LRR-Luc* construct (Figure 4B–C, Table S3) while the remaining deletions did not affect either light-induced expression or the phase of rhythmic expression (Figure S4 and Table S3) driven by the *cry1a* LRR. Located in the region of sub-deletions 5 and 6 (see red dotted lines and arrowheads in Figure 4A) is a single D-box element (5′-GTTGTATAAC-3′) with a distinct sequence from that of the functional D-box identified in the *per2* promoter (5′-CTTATGTAAA-3′) [21]. Mutation of this D-box within the *cry1a* LRR results in the complete disruption of the characteristic light inducible expression pattern of the *cry1a LRR-Luc* construct (Figure 4D, Tables S3 and 4), similar to the results obtained with sub-deletions 5 and 6 (see Figure 4B–C).

Is this *cry1a* D-box element sufficient to confer a light-regulated pattern of expression? To address this question, we analyzed the expression of a heterologous construct containing multimerized repeats of this D-box cloned upstream of a minimal promoter and luciferase reporter (*D-box_cry1a_-Luc*) (Figure 4E). Similar to the functional D-box located within the *per2* promoter [21], the D-box within the *cry1a* LRR shows a clear light-driven rhythmic pattern of expression with an increase after “lights on” and arhythmicity after transfer of the cells into DD conditions. This data demonstrates that this D-box alone is sufficient to direct a light inducible expression pattern that closely resembles that of the *cry1a* promoter. Thus, the light regulation of the *cry1a* gene appears to be mediated by a single D-box enhancer element.

### Light Induced D-box Enhancer Activity Requires *de novo* Protein Synthesis

Given that light–induced expression of both *per2* and *cry1a* is dependent on the D-box enhancer, we speculated whether the differential requirement of the two genes for protein synthesis may be linked to differences in the sequences of the two D-boxes in their promoters or alternatively to the contribution of the *per2* E-box enhancer. To test these hypotheses we treated with CHX, PAC-2 cells transfected with various heterologous E- and D-box luciferase reporter constructs. Then, luciferase mRNA expression was monitored by qRT-PCR analysis following exposure to light.

**Figure 5:**
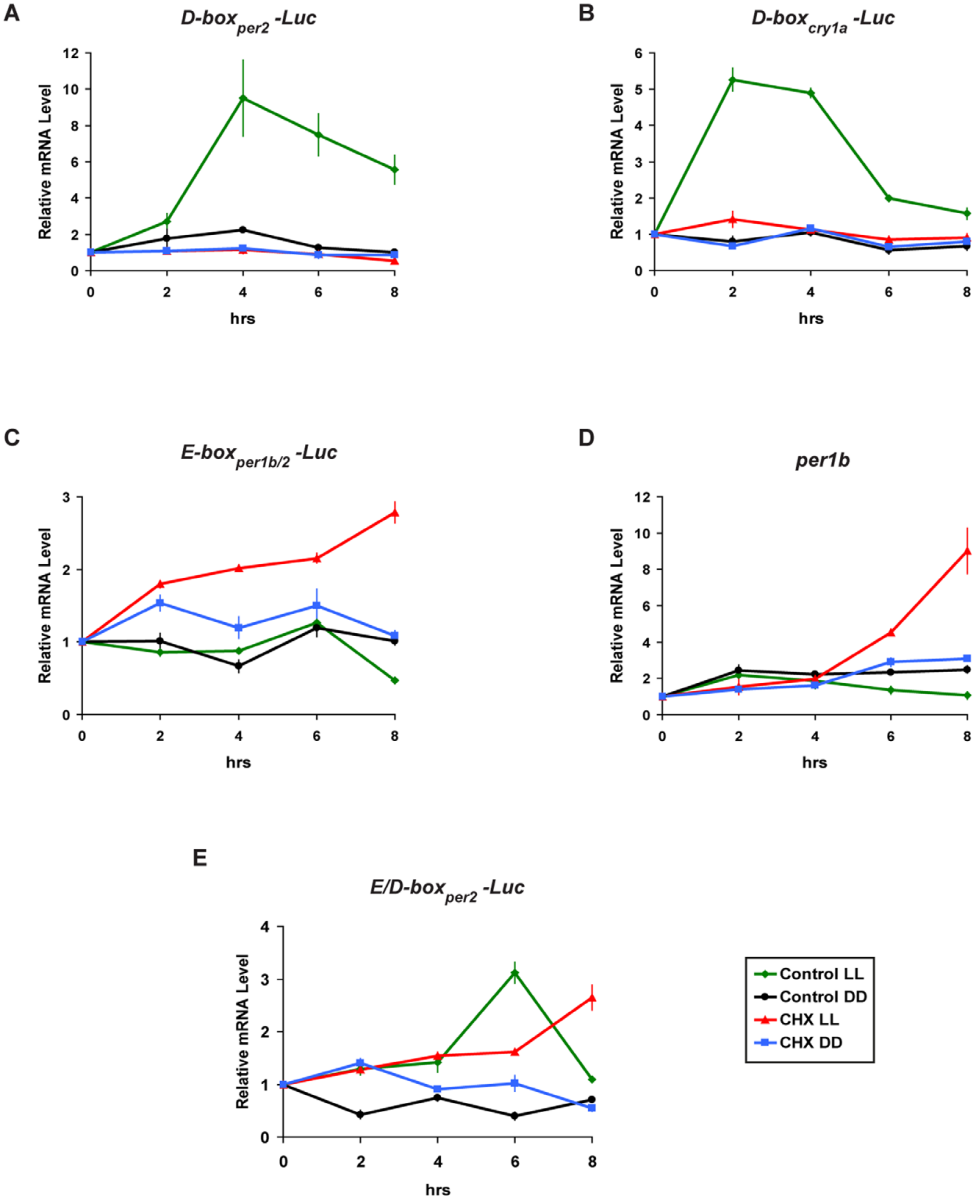
Light induced D-box enhancer activity requires *de novo* protein synthesis. (A–C and E) qRT-PCR analysis of luciferase mRNA expression in PAC-2 cells transfected with different heterologous luciferase reporter constructs, in the presence (red traces) or absence (green traces) of CHX during 8 hours of light exposure or DD conditions (+CHX, blue traces, −CHX, black traces). (D) qRT-PCR analysis of endogenous *per1b* expression in PAC-2 cells in the presence or absence of CHX during 8 hours of light exposure or DD conditions (colour coded the same as in panels A–C and E). Each construct is indicated above its respective panel. Relative mRNA levels are plotted on the y-axis and were set arbitrarily as 1 at time-point 0 hrs. Time (hrs) is plotted on the x-axis. In each panel, points are plotted as means of three independent experiments +/− SD. All statistical analyses (two-way ANOVA) are presented in Table S2 B or in the results section.

Neither the *D-box_per2_ -Luc* nor the *D-box_cry1a_ -Luc* construct was able to drive light induced luciferase mRNA expression when protein synthesis was blocked by CHX treatment (Figure 5A–B and Table S2 B). In contrast, upon light exposure expression of the transfected E-box reporter (*E-box_per1b/2_-Luc*) as well as the endogenous E-box-regulated gene, *per1b*, displayed a progressive increase that persisted for the entire time course of CHX treatment, compared with non treated and constant dark controls (Figure 5C,D and Table S2 B). The light-induced gene expression driven by E-boxes during CHX treatment exhibit different kinetics compared with those of the D-box under normal conditions (p<0.0001, two-way ANOVA). Finally, the expression of a luciferase reporter construct containing tandem repeats of adjacent E- and D-boxes (*E/D-box_per2_-Luc*), showed a light induced expression pattern in the presence or absence of CHX (Figure 5E and Table S2 B). Thus, this artificial construct based on the structure of the light responsive module of the *per2* promoter [21], behaves in a similar fashion to the endogenous *per2* gene (see Figure 1A and Table S2 B). Together these results indicate that it is the E-box within the *per2* promoter that confers the specific light dependent response of the *per2* gene in the absence of *de novo* protein synthesis. The more general requirement of light induced gene expression for protein synthesis appears to reflect the regulatory properties of the D-box enhancer element.

### Regulation of the *cry1a* D-box by PAR bZip Transcription Factors

**Figure 6:**
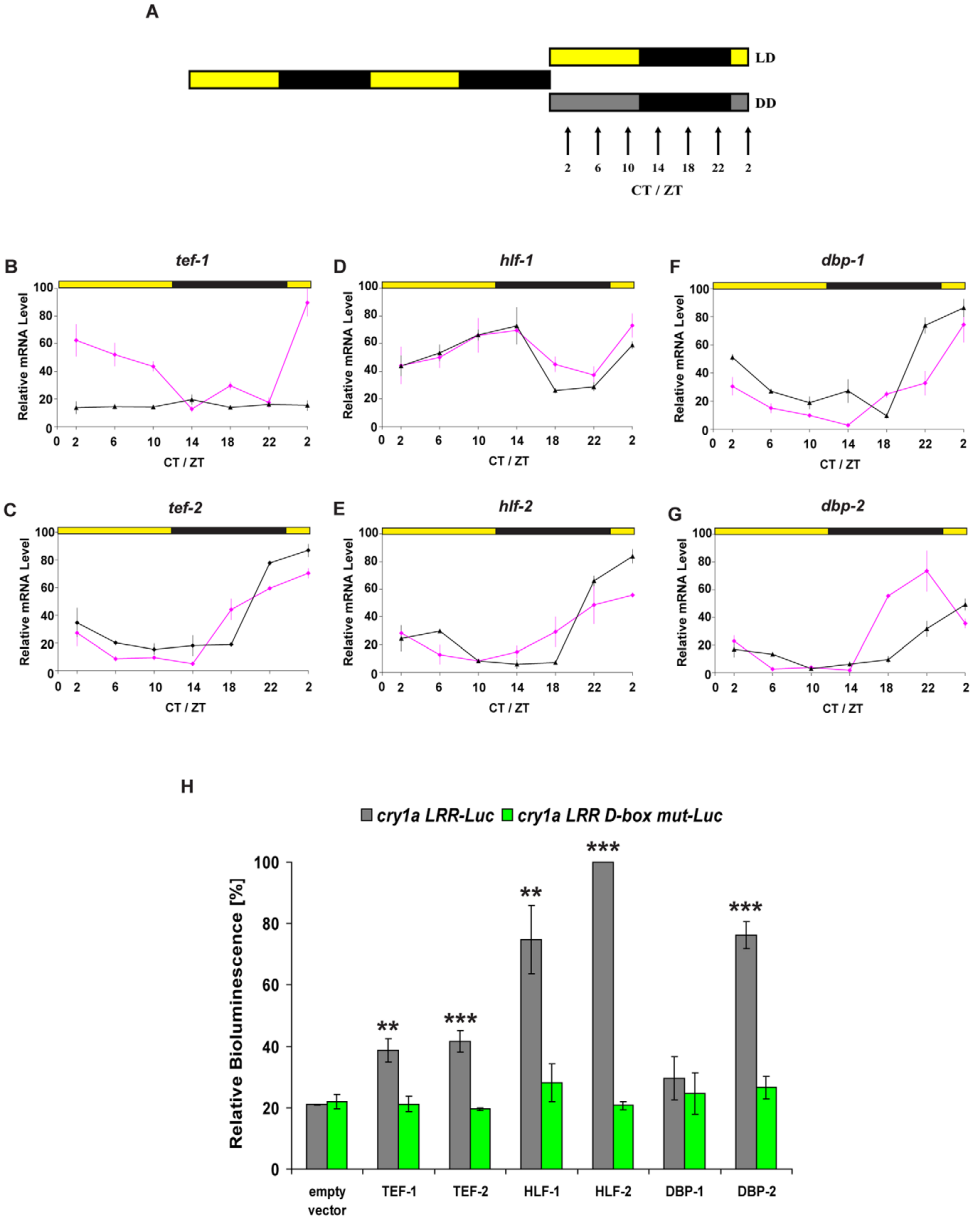
Regulation of the *cry1a* D-box by PAR bZip transcription factors. (A) Schematic representation of the experimental design. Black and yellow bars represents 12 hours dark and light periods respectively, while the dark grey bar denotes the subjective day period under constant darkness. Arrows indicate sampling time points where ZT and CT represent zeitgeber times and circadian times respectively (ZT0 represents “lights on”). (B–G) qRT-PCR analysis of PAR bZip gene expression in PAC-2 cells under LD (pink traces) and DD (black traces) conditions. Each gene is indicated above its respective panel. Relative mRNA levels are plotted on the y-axis and ZT or CT times on the x-axes. In each panel, points are plotted as the means of three independent experiments +/− SD. Yellow and black bars above each panel represent the light and dark periods, respectively. The statistical significance of rhythmic expression was assessed by t-test analysis in Table S2 C. (H) *In vitro* luciferase assay of PAC-2 cells co-transfected with expression constructs encoding the six PAR bZip factors and the *cry1a LRR-Luc* or *cry1a LRR D-box mut-Luc* reporters (dark grey and green bars, respectively). Each expression construct is indicated below its respective bars. Relative bioluminescence levels (%) are plotted on the y-axis where the highest value measured during the experiment is set arbitrarily as 100%. The results are plotted as the means of three independent experiments performed in triplicate, +/− SD. Each independent experiment was standardized for transfection efficiency using a β-galactosidase assay. The statistical significance of levels of transactivation was assessed by t-test analysis with * p<0.05, ** p<0.001, *** p<0.0001.

Which factors play a role in the protein synthesis-dependent activation of *cry1a* by light? We have recently identified a family of zebrafish transcription factors which share homology with the mammalian D-box binding PAR bZip transcription factors DBP, TEF and HLF [21], [24]. These factors exhibit both clock and light driven expression in a range of zebrafish embryonic tissues [21], [24]. We first wished to study the clock and light regulated expression of these factors in PAC-2 cells. Cells were entrained for 2 days to LD cycles and then subsequently harvested at regular time points on the third day either under LD or constant darkness conditions for subsequent qRT-PCR analysis (Figure 6A–G). With the exception of TEF-1, all the PAR bZip factors exhibited robust rhythms of expression both under LD and DD conditions pointing to strong circadian clock regulation (Figure 6C–G and Table S2 C). In contrast, for TEF-1, while rhythmic expression was evident under LD conditions, constant expression was detected during the first day in constant darkness (Figure 6B and Table S2 C). Thus, while TEF-1 appears to behave as a predominantly light driven gene (see also Figure 1B), all other PAR bZip factors are dynamically expressed under LD cycles. Which members of this family are able to mediate transcriptional transactivation by the D-box within the *cry1a* LRR region? To address this question we performed an *in vitro* luciferase assay testing the effect of co-expression of each PAR bZip factor with the *cry1a LRR-Luc* reporter (Figure 6H). All the factors analysed with the exception of DBP-1 activated reporter gene expression although with different levels of induction (dark grey bars). Consistent with this activation by the PAR bZip factors being D-box mediated, mutation of this D-box element within the *cry1a* LRR completely abolished the observed transactivation (green bars). Thus, it is tempting to speculate that a requirement for expression of the zebrafish PAR bZip factors could potentially explain the protein synthesis dependence of D-box-mediated transcription.

## Discussion

Teleosts have proved to be fascinating models for exploring how light regulates the vertebrate circadian timing system [10]. However, light exposure of most tissues and cells triggers expression of a set of genes that is not restricted to components of the circadian clock [19], [20], [24]. Thus, a key question is how diverse the regulatory mechanisms are which link photoreceptors with gene expression. We have now demonstrated that the D-box serves as the principle light responsive promoter element in both light inducible zebrafish clock genes. Together with a general enrichment of D-box enhancers in the promoters of light induced genes [19], this implies a general importance of this element in light responsive transcription. In mammals, D-box binding factors appear to play a key role linking the circadian clock mechanism with downstream targets [27]. Thus, our findings suggest that during vertebrate evolution, there has been a major shift in the role of D-boxes from being the targets of light signaling pathways to being elements of clock output pathways. In this regard it will be of great interest to compare the role of D-boxes in mammals and teleosts in other physiological mechanisms.

**Figure 7:**
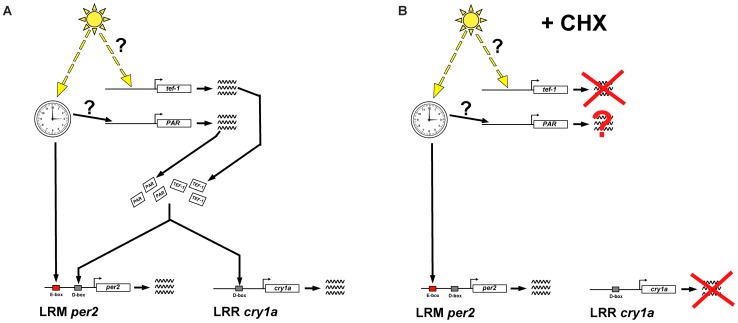
Contribution of *de novo* protein synthesis to light–induced clock gene expression. (A) Under normal conditions light exposure triggers expression of the gene encoding the PAR bZip factor, TEF-1. This in turn binds to D-boxes in the *cry1a* and *per2* promoters and trans-activates gene expression. In parallel, light also entrains the circadian clock. Via binding of the CLOCK–BMAL complex, the clock regulates the E-box in the *per2* promoter and thereby contributes to light induced gene expression [21]. The clock also regulates expression of the additional PAR bZip factors (PAR) that contribute to D-box driven transcription. (B) Upon light exposure and coincident inhibition of *de novo* protein synthesis by treatment with cycloheximide (+CHX), translation of TEF-1 and the other PAR bZip factors is prevented. Therefore, light-driven transactivation via the D-box enhancer of the *cry1a* promoter is abolished. However, light-induced expression of the *per2* promoter persists due to regulation by the E-box. Specifically, upon cycloheximide treatment the core clock machinery directs increased activation via the E-box in a light dependent manner. We speculate that this up-regulation of E-box driven expression may also influence other clock-regulated genes including those encoding the PAR bZip factors.

Our results (summarized in Figure 7) reveal that D-box – mediated light-induced gene expression requires *de novo* protein synthesis. Our previous studies have demonstrated that a family of 6 PAR bZip D-box binding factors is widely expressed in zebrafish tissues [21], [24]. Many of these genes are clock regulated and the expression of one member of this family, *tef-1* is directly induced by light. Here we demonstrate that these factors show similar clock or light driven regulation in the PAC-2 cell line. Furthermore, with one possible exception (DBP-1), all factors serve to trans-activate expression from the D-box located in the cry1a LRR. We therefore speculate that translation of these transcription factors may be a prerequisite for D-box function. Given that *tef-1* is encoded by a light-inducible gene, it is tempting to speculate that it may play a preferential role in relaying lighting information to the regulation of gene expression. However, the kinetics of light induced *tef-1* mRNA expression is very similar to that of other light induced genes (see Figure 1). Specifically, it does not display the rapid induction characteristic of immediate early response genes that one would predict for an upstream element. Furthermore, while the activation of immediate early response genes classically does not rely upon *de novo* protein synthesis, we have shown that light induced mRNA expression of *tef-1* is blocked by cycloheximide treatment. This implies that upstream of *tef-1* there may be additional light-regulated, immediate early response regulators. The original transcriptome analysis which lead to identification of many light-induced genes was based on a commercial microarray and therefore is far from representative of the entire light regulated transcriptome [19]. For this reason it will be valuable to use whole transcriptome sequencing approaches to search more systematically for immediate early response genes that might lie upstream of D-box regulators.

Amongst immediate early response genes, those encoding elements of the AP-1 transcription factor complex such as *c-fos* and *c-jun* have been well documented to rapidly relay changes in the cellular environment to gene expression [28], [29]. Interestingly, in a previous report, light-dependent changes in AP-1 DNA binding activity were implicated as playing a key role in the response of the zebrafish *cry1a* gene to light [25]. However, our results failed to confirm these predictions. In the previous study, the contribution of AP-1 sites in the *cry1a* promoter was not tested by a functional promoter analysis. Furthermore, the Z3 cell line used in that study was derived independently from the PAC-2 cell line and so may exhibit different regulatory properties [30]. Thus, in the future it will clearly be important to assess how light dependent changes in AP-1 DNA binding may interact with the D-box transcriptional regulatory machinery, possibly in a promoter dependent fashion.

We have demonstrated that the continued induction of the *per2* gene by light in the absence of protein synthesis is based on regulation by the E-box element within the light responsive module of its promoter (Figure 7). Given that the E-box represents a target of the core circadian clock mechanism, this implies that light induced *per2* expression requires regulatory input from the circadian clock. The apparent absence of this regulation in the *cry1a* gene implies basic differences in the roles for these two negative elements of the core clock mechanism. Interestingly, the basal level of E-box driven expression increases in a light-dependent fashion in the absence of protein synthesis. It is tempting to speculate that this may result from the lack of synthesis of negative regulators of CLOCK and BMAL upon light exposure. Alternatively, this may reflect the existence of light-dependent post-translational modifications to the core clock elements that modulate E-box function.

Previous studies have identified many potential candidates for the photoreceptors in zebrafish peripheral tissues including opsins, cryptochromes and ROS [18], [22], [23]. The finding that D-boxes serve as the principle light responsive enhancer elements therefore predicts that D-box binding transcription factors may serve as key convergence points for diverse light dependent signaling pathways.

## Materials and Methods

### Cell Culture

The PAC-2 cell line [31] was cultured as previously described [15], [32]. For incubation under different lighting regimes, cells were maintained in thermostatically controlled darkrooms or light-sealed incubators and were illuminated with a tungsten light source (20 µW/cm^2^). Cycloheximide (CHX), puromycin, anisomycin and phorbol ester 12-O-tetradecanoylphorbol-13-acetate (TPA) treatments were as recommended by the manufacturer (Sigma Aldrich). Stable PAC-2 cell lines were established as described elsewhere [15], [21]. The FuGene HD reagent was used for transient transfections according to the manufacturer’s protocol (Roche Diagnostics).

### Quantitative RT-PCR (qRT-PCR)

Total RNA was extracted using Trizol reagent (GIBCO-BRL) according to the manufacturer’s instructions. Total RNA was reverse-transcribed into cDNA by using Superscript III Reverse Transcriptase (Invitrogen) with a mixture of oligo dT and random primers. Quantitative RT-PCR analysis was performed using a StepOnePlus Real-Time RT-PCR System (Applied Biosystems) and SYBR Green I fluorescent dye (Qiagen). Relative expression levels were normalized using zebrafish β*-actin*. The relative levels of each mRNA were calculated using the 2-ΔΔCT method. For each gene the primer sequences used for qRT-PCR are listed in Table S1.

### Luciferase Constructs

#### cry1a-Luc

A DNA fragment of 1.3 kb encompassing 1.25 kb of the 5′ flanking and 53 bp of exon 1 of the *cry1a* gene was PCR amplified from zebrafish genomic DNA using specific primers incorporating a 5′ KpnI (5′–GACCACAGACTGGTACCGTGCATTAAA–3′) and a 3′ XhoI restriction site (5′–AGATCTCGAGGCCGCAAGCCCTTCCTG–3′) using a XL PCR kit (Roche). The PCR product was then cloned into the luciferase expression vector pGL3Basic (Promega).

#### cry1a LRR-Luc

The 186 bp light responsive region (LRR) identified in the *cry1a* promoter was PCR amplified from the *cry1a-Luc* construct with the following primers: 5′ KpnI (5′–GCATAACTCGGTACCCAACTTTCTCTACATGCGAG–3′) and 3′ XhoI (5′–AATTTGGAACTCGAGCACAGATGAAGC–3′) and cloned into pGL3Basic.

#### cry1a-Luc deletion 1 to 17 and cry1a LRR-Luc sub-deletion 1 to 13

Luciferase constructs were generated using as template the *cry1a-Luc* and the *cry1a LRR-Luc* constructs, respectively by a PCR based deletion strategy as previously described [21]. The exact position and length of each deletion with respect to the ATG (position +1) is listed in Table S5. Period (τ) and peak (ZT) values for all deletion constructs are listed in Table S3.

### Mutagenesis

Site directed mutagenesis was performed using the QuikChange Multi Site-Directed Mutagenesis Kit (Stratagene) according to the manufacturer’s instructions. Table S4 displays the primer sequences containing the specific mutations introduced for each construct.

### Heterologous Constructs

All heterologous promoter constructs were based on the minimal promoter luciferase expression vector pLucMCS (Stratagene). ***E-box_per1b/2_-Luc*** contains four copies of the *per1b* E-box (5′–CACGTG–3′) [15] which is identical to that in the *per2* gene LRM region [21]. ***D-box_per2_-Luc*** contains six copies of the *per2* D-box 5′–CTTATGTAAA–3′ [21]. ***D-box_cry1a_-Luc*** contains four copies of the *cry1a* D-box 5′–AAGTTATACAAC–3′ (position −331 bp relative to the ATG). ***E/D-box_per2_-Luc*** reporter construct contains four copies of alternating *per2* E-box (5′–CACGTG–3′) and D-box (5′–CTTATGTAAA–3′) sequences [21]. Finally, the ***AP1-Luc*** reporter construct consists of four copies of the sequence 5′-TGACTCA-3′ (canonical *cry1a* AP-1 #1 site, Figure 2A).

### Real-Time Bioluminescence Assay and Data Analysis

All real-time bioluminescence assays were performed and analyzed as described previously [15], [21] using an EnVision multilabel counter (Perkin Elmer) under various lighting conditions.

### Expression Constructs

All PAR bZip factor expression constructs (TEF-1, TEF-2, HLF-1, HLF-2, DBP-1, DBP-2) were based on the CMV promoter driven expression vector pCS2-MTK. Identification and cloning of all six PAR transcription factor cDNAs is described elsewhere [21], [24]. The N-terminally myc-tagged Cry1a expression construct which was used in the experiment presented in Figure S1 A, was based on pCS2-MTK.

### *In Vitro* Luciferase Assay

PAC-2 cells were plated at a density of 1.25×10^5^ cells per well in a 24-well plate (CELLSTAR, Greiner Bio-One). 24 hours later, cells were cotransfected with 250 ng of *cry1a LRR-Luc* or *cry1a LRR D-box mut-Luc* reporters, 50 ng for each PAR bZip expression vector together with 50 ng of β-galactosidase expression vector (to normalize for transfection efficiency). All transfections were performed using FuGene HD reagent according to the manufacturer’s recommendations (Roche Diagnostics). β-galactosidase activity assays were performed using a standard protocol [33]. Luciferase activity was measured using the Luciferase Assay System kit (Promega) and a VICTOR Multilabel Plate Reader (Perkin Elmer) following the manufacturer’s instructions.

### Western Blotting

Protein extracts were prepared by homogenizing samples in 1×Laemmli buffer. The samples were electrophoresed on a SDS polyacrylamide gel and transferred to an Immobilon-P membrane (Millipore). Binding of the antibodies was visualized using the Pierce-ECL detection system (Thermo Scientific). The myc antibody was purchased from Santa Cruz and β-actin antibody from Sigma Aldrich.

### Statistical Analysis

Data were analyzed by unpaired t-test and two-way ANOVA using GraphPad Prism 4.0 for Windows (Graph Pad Software, http://www.graphpad.com). All the results were expressed as means +/− SD. p<0.05 was considered statistically significant. For all t-tests presented in Table S2 A (referring to Figure 1) the values at “time 0″ and the highest values observed in the time course of each experiment were considered. For all t-tests presented in Table S2 C (referring to Figure 6B–G) the peak and trough values observed under LD or DD conditions were considered. Period (τ) and peak (ZT) values in Table S3 were calculated using Cosinor analyses performed using COSINOR v3.0.2 software (Antoni Diez-Noguera, University of Barcelona).

## Supporting Information

Figure S1 **(A)** **Cycloheximide effectively blocks protein synthesis in PAC-2 Cells.** Representative data from western blotting analysis of PAC-2 cells transiently transfected with a myc-tagged Cry1a expression vector. 18 hrs after transfection the cells were treated with CHX (10 µg/ml) or vehicle (DMSO) and then harvested for protein extracts during a 36 hours time course. Myc-tagged protein and endogenous beta-actin protein levels were visualized. **(B)** **Endogenous** ***β-actin*** **mRNA levels are not affected by cycloheximide or light treatment.** qRT-PCR analysis of endogenous *β-actin* mRNA expression in PAC-2 cells in the presence (red traces) or absence (black traces) of CHX during 8 hours of light exposure (left panel) or under DD conditions (right panel). The samples analyzed were those tested in Figure 1. Yellow and black bars above each panel indicate the lighting conditions. Relative mRNA levels are plotted on the y-axis and were set arbitrarily as 1 at time-point 0 hrs. Time (hrs) is plotted on the x-axis. In both panels, points are plotted as means of four independent experiments +/− SD.
(TIF)

Figure S2 **Effect of alternative protein synthesis inhibitors.** qRT-PCR analysis of endogenous *per2* and *cry1a* expression in PAC-2 cells in the presence (red traces) or absence (black traces) of either (A) puromycin or (B) anisomycin during 8 hours of light exposure. After 3 days in DD the cells were treated with either puromycin (35 µM) or anisomycin (35 µM) 1 h before sampling. Yellow bars above each panel indicate the lighting conditions. Relative mRNA levels are plotted on the y-axis and were set arbitrarily as 1 at time-point 0 hrs. Time (hrs) is plotted on the x-axis. In each panel, points are plotted as means of three independent experiments +/− SD.
(TIF)

Figure S3 ***cry1a-Luc*** **deletion constructs analysis.** Representative real time bioluminescence assay of PAC-2 cells transfected with *cry1a-Luc* (black trace) or *cry1a-Luc deletion* constructs (green traces) under different lighting conditions. Each construct is indicated above its respective panel. In each panel relative bioluminescence is plotted on the y-axis and time (hrs) on the x-axis. Each time-point represents the mean of at least four independently transfected wells +/− SD from a single experiment. Each experiment was performed a minimum of three times. Yellow and black bars above each panel represent the light and dark periods, respectively.
(TIF)

Figure S4 ***cry1a LRR-Luc*** **sub-deletion constructs analysis.** Representative real time bioluminescence assay from PAC-2 cells transfected with *cry1a LRR-luc* (black trace) or *cry1a LRR-luc sub-deletion* constructs (green traces) under different lighting conditions. Each construct is indicated above its respective panel. In each panel relative bioluminescence is plotted on the y-axis and time (hrs) on the x-axis. Each time-point represents the mean of at least four independently transfected wells +/− SD from a single experiment. Each experiment was performed a minimum of three times. Yellow and black bars above each panel represent the light and dark periods, respectively.
(TIF)

Table S1 **qRT-PCR primer sequences.**
(DOC)

Table S2 **t-test and two-way ANOVA analysis.** (A) t-test analysis of data presented in Figure 1. In all these t-tests, the values at “time 0″ and the highest values observed in the time course of each experiment are considered. (B) Two-way ANOVA analysis of data presented in Figures 1 and 5. Lighting conditions are indicated by colour-coding (yellow for light exposure and dark grey for constant darkness). Cycloheximide treatment is indicated by +CHX and non-treated controls by −CHX. (C) t-test analysis of data presented in Figure 6B–G. In all these t-tests, the values obtained at the peaks and troughs were considered. In all three panels, “N.S.” denotes no statistical significance (p>0.05).
(DOC)

Table S3 **Cosinor analysis.** Period (τ) and Peak (ZT) values for all luciferase reporter constructs analyzed. “N.S.” denotes no statistically significant rhythm detected (p>0.05).
(DOC)

Table S4 **Mutagenesis primer sequences.** Wild type target sequences are indicated and the mutated counterparts are highlighted in bold in the sequences.
(DOC)

Table S5 **Position and size of all deletions and sub-deletions generated in the context of** ***cry1a-Luc*** **and** ***cry1a LRR-Luc,*** **respectively.**
(DOC)
