# Supplementary figures and images for: Regulation of per and cry Genes Reveals a Central Role for the D-Box Enhancer in Light-Dependent Gene Expression
Source: PLoS One. 2012 Dec 6;7(12):e51278. doi: 10.1371/journal.pone.0051278 (PMC3516543; doi:10.1371/journal.pone.0051278)

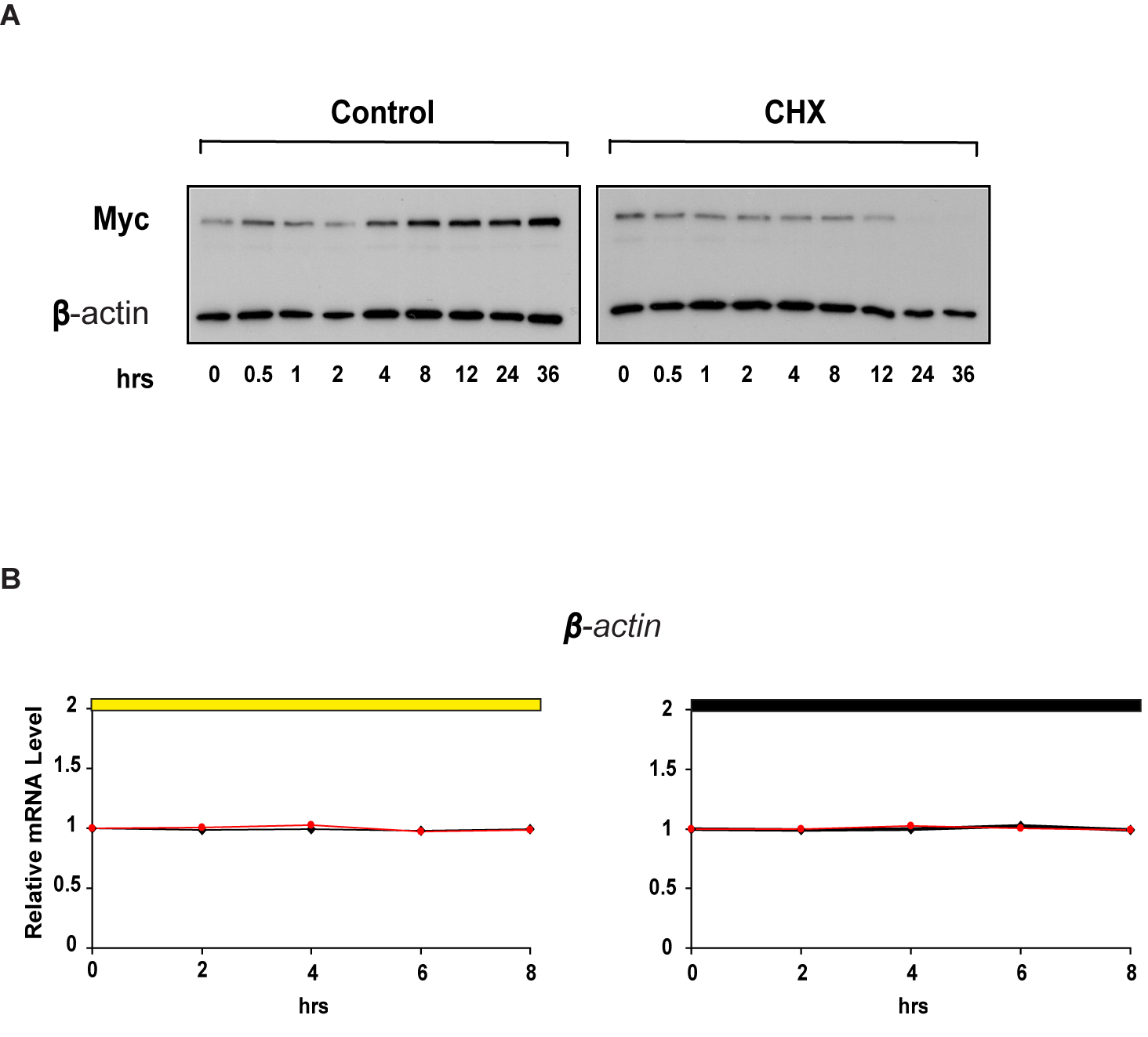

Supplement: Figure S1 — (A) Cycloheximide effectively blocks protein synthesis in PAC-2 Cells. Representative data from western blotting analysis of PAC-2 cells transiently transfected with a myc-tagged Cry1a expression vector. 18 hrs after transfection the cells were treated with CHX (10 µg/ml) or vehicle (DMSO) and then harvested for protein extracts during a 36 hours time course. Myc-tagged protein and endogenous beta-actin protein levels were visualized. (B) Endogenous β-actin mRNA levels are not affected by cycloheximide or light treatment. qRT-PCR analysis of endogenous β-actin mRNA expression in PAC-2 cells in the presence (red traces) or absence (black traces) of CHX during 8 hours of light exposure (left panel) or under DD conditions (right panel). The samples analyzed were those tested in Figure 1. Yellow and black bars above each panel indicate the lighting conditions. Relative mRNA levels are plotted on the y-axis and were set arbitrarily as 1 at time-point 0 hrs. Time (hrs) is plotted on the x-axis. In both panels, points are plotted as means of four independent experiments +/− SD. (TIF) [file pone.0051278.s001.tif]

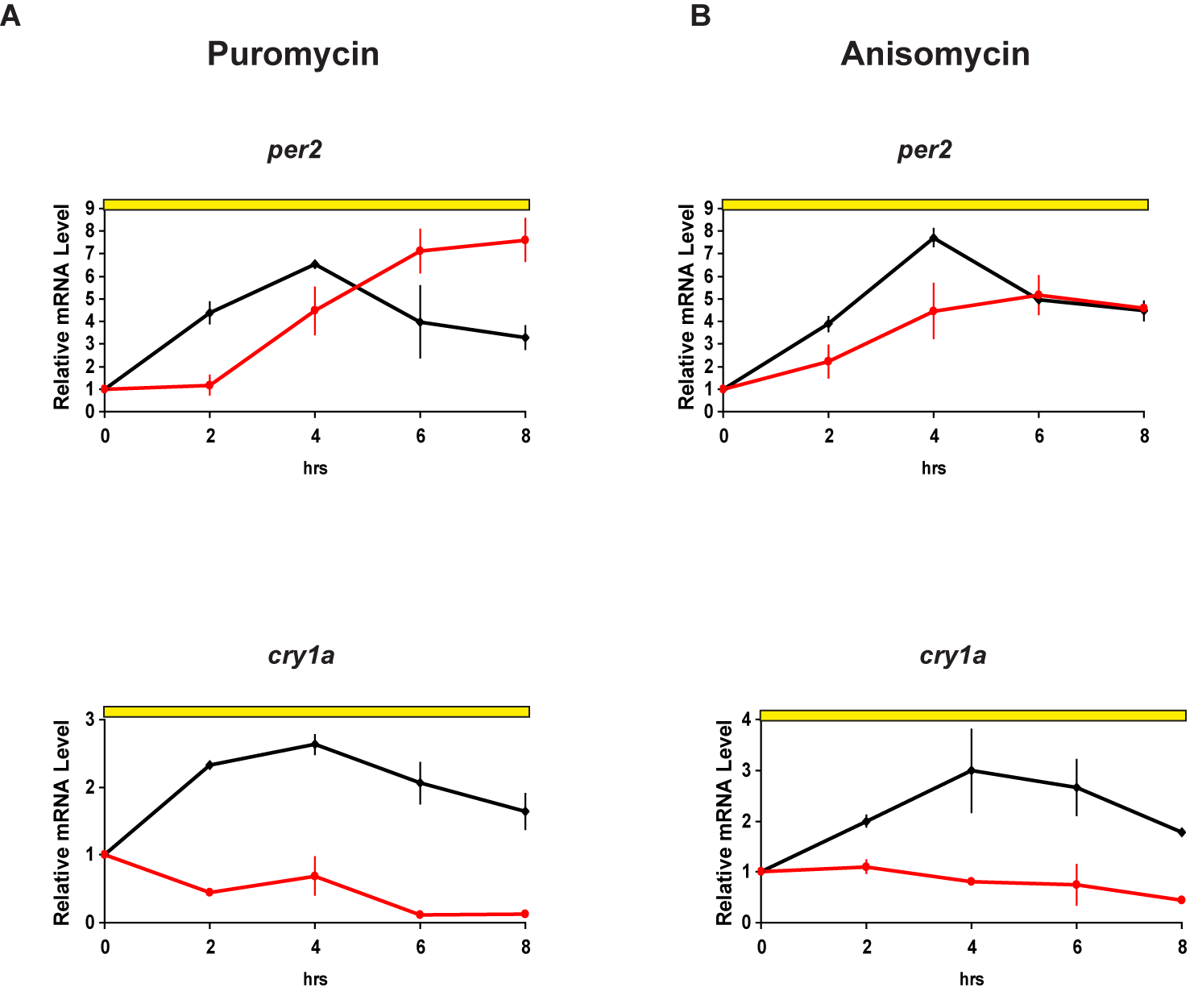

Supplement: Figure S2 — Effect of alternative protein synthesis inhibitors. qRT-PCR analysis of endogenous per2 and cry1a expression in PAC-2 cells in the presence (red traces) or absence (black traces) of either (A) puromycin or (B) anisomycin during 8 hours of light exposure. After 3 days in DD the cells were treated with either puromycin (35 µM) or anisomycin (35 µM) 1 h before sampling. Yellow bars above each panel indicate the lighting conditions. Relative mRNA levels are plotted on the y-axis and were set arbitrarily as 1 at time-point 0 hrs. Time (hrs) is plotted on the x-axis. In each panel, points are plotted as means of three independent experiments +/− SD. (TIF) [file pone.0051278.s002.tif]

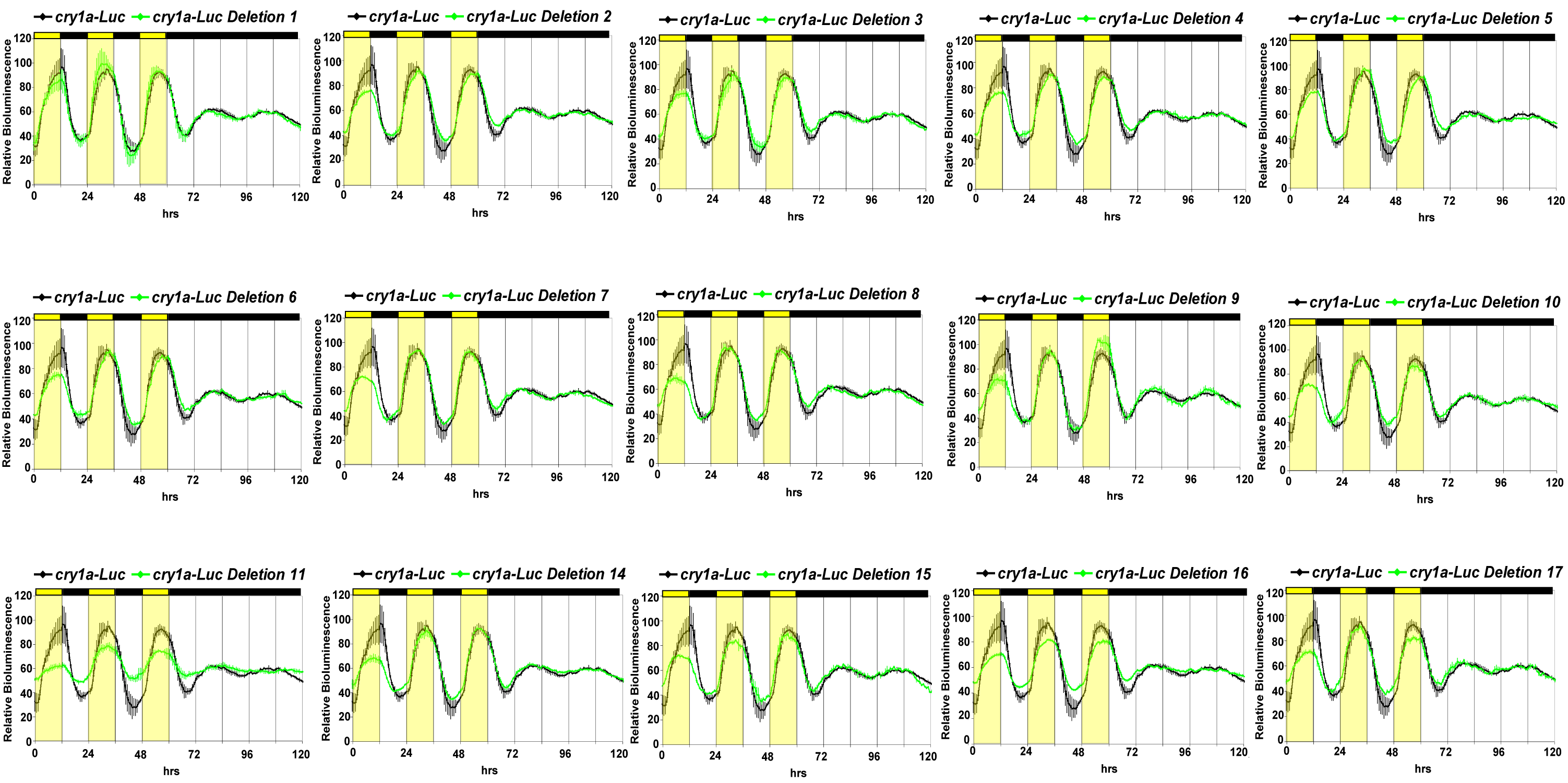

Supplement: Figure S3 — cry1a-Luc deletion constructs analysis. Representative real time bioluminescence assay of PAC-2 cells transfected with cry1a-Luc (black trace) or cry1a-Luc deletion constructs (green traces) under different lighting conditions. Each construct is indicated above its respective panel. In each panel relative bioluminescence is plotted on the y-axis and time (hrs) on the x-axis. Each time-point represents the mean of at least four independently transfected wells +/− SD from a single experiment. Each experiment was performed a minimum of three times. Yellow and black bars above each panel represent the light and dark periods, respectively. (TIF) [file pone.0051278.s003.tif]

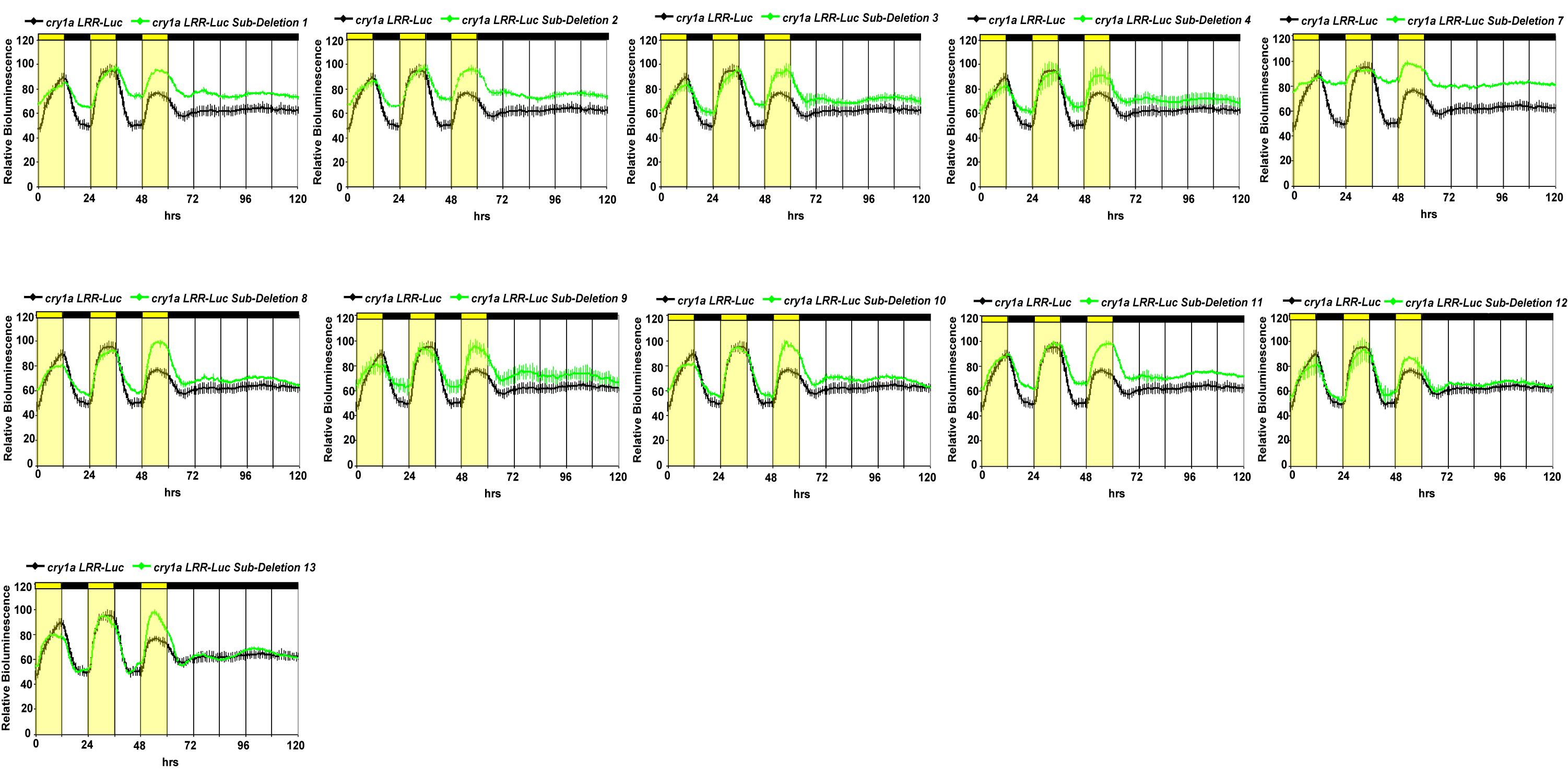

Supplement: Figure S4 — cry1a LRR-Luc sub-deletion constructs analysis. Representative real time bioluminescence assay from PAC-2 cells transfected with cry1a LRR-luc (black trace) or cry1a LRR-luc sub-deletion constructs (green traces) under different lighting conditions. Each construct is indicated above its respective panel. In each panel relative bioluminescence is plotted on the y-axis and time (hrs) on the x-axis. Each time-point represents the mean of at least four independently transfected wells +/− SD from a single experiment. Each experiment was performed a minimum of three times. Yellow and black bars above each panel represent the light and dark periods, respectively. (TIF) [file pone.0051278.s004.tif]
