# Supplementary material for: Regulation of per and cry Genes Reveals a Central Role for the D-Box Enhancer in Light-Dependent Gene Expression
Source: PLoS One. 2012 Dec 6;7(12):e51278. doi: 10.1371/journal.pone.0051278 (PMC3516543; doi:10.1371/journal.pone.0051278)
Supplement: Table S1 — qRT-PCR primer sequences. (DOC) [file pone.0051278.s005.doc]

**Supplementary Table 1**

**qRT-PCR Primers**

| **Gene** | **Primer Sequence** |
| --- | --- |
| *per2* | Forward: 5’- GCTTCACCACACCATACAGG -3’  Reverse: 5’- GTCTGACGGGGACGAGTCT -3’ |
| *cry1a* | Forward : 5’- TCCGCTGTGTGTACATCCTC -3’  Reverse : 5’- CAAACACTGCAGCAAAAACC -3’ |
| *tef-1* | Forward: 5’- AAAGCTCTGCTTGAGTACCCTTT -3’  Reverse: 5’- ACTCCTCCAGATCCATGTACTCC -3’ |
| *e4bp4-6* | Forward: 5’- ACCTTCAGCGCAGCATTACT -3’  Reverse: 5’- CTCGATGTGGGGACAGTTTT -3’ |
| *6-4photolyase* | Forward: 5’- AATGGCAAGACTCCCATGAC -3’  Reverse: 5’- GTGGCCCTAAGGATGACGTA -3’ |
| *lonrf1(2of2)* | Forward: 5’- TGCATTTTATTTGAAAGTTGACA -3’  Reverse: 5’- ATTTGTCATTCGCCTCTTGG -3’ |
| *tef-2* | Forward: 5’- TGAGCGTGCTACACCATCTC -3’  Reverse: 5’- CCTTCTTGATCATGGGCTGT -3’ |
| *hlf-1* | Forward: 5’- ATTGACCCAGACTCCATCCA -3’  Reverse: 5’- CCTTCTTGATCATGGGCTGT -3’ |
| *hlf-2* | Forward: 5’- CAACAACAGCAGCAACAGGT -3’  Reverse: 5’- GATCGAAAACCTCCTGTCCA -3’ |
| *dbp-1* | Forward: 5’- GTCCAGGCCAATTTCTCAAA -3’  Reverse: 5’- CACAGCAGTCCTTCCCTCTC -3’ |
| *dbp-2* | Forward: 5’- AAGATGCTCGTCCCTGAAGA -3’  Reverse: 5’- CCAGATGGTGGCTCTCGTAT -3’ |
| *per1b* | Forward: 5’- ATGTGCAGGCTGTAGATCCC -3’  Reverse: 5’- CCGTCAGTTTCGCTTTTCTC -3’ |
| *luciferase* | Forward: 5’- ATGAAGAGATACGCCCTGGTT -3’  Reverse: 5’- CTGCATACGACGATTCTGTGA -3’ |
| * β-actin* | Forward : 5’- GCCTGACGGACAGGTCAT -3’  Reverse : 5’- ACCGCAAGATTCCATACCC -3’ |
