# Supplementary material for: Regulation of per and cry Genes Reveals a Central Role for the D-Box Enhancer in Light-Dependent Gene Expression
Source: PLoS One. 2012 Dec 6;7(12):e51278. doi: 10.1371/journal.pone.0051278 (PMC3516543; doi:10.1371/journal.pone.0051278)
Supplement: Table S2 — t-test and two-way ANOVA analysis. (A) t-test analysis of data presented in Figure 1. In all these t-tests, the values at “time 0″ and the highest values observed in the time course of each experiment are considered. (B) Two-way ANOVA analysis of data presented in Figures 1 and 5. Lighting conditions are indicated by colour-coding (yellow for light exposure and dark grey for constant darkness). Cycloheximide treatment is indicated by +CHX and non-treated controls by −CHX. (C) t-test analysis of data presented in Figure 6B–G. In all these t-tests, the values obtained at the peaks and troughs were considered. In all three panels, “N.S.” denotes no statistical significance (p>0.05). (DOC) [file pone.0051278.s006.doc]

**Supplementary Table 2**

**A B**

|  | **two-way ANOVA** | | |
| --- | --- | --- | --- |
| **Gene** | **- CHX vs. + CHX** | **- CHX vs. + CHX** | **+ CHX vs. + CHX** |
| *per2* | N.S. | N.S. | p < 0.0001 |
| *cry1a* | p < 0.0001 | N.S. | N.S. |
| *tef-1* | p < 0.0001 | N.S. | N.S. |
| *e4bp4-6* | p < 0.0001 | N.S. | N.S. |
| *6-4photolyase* | p < 0.0001 | N.S. | N.S. |
| *lonrf1(2of2)* | p < 0.0001 | N.S. | N.S. |
| *per1b* | p < 0.0001 | N.S. | p < 0.0001 |
| *D-box_per2_ -Luc* | p < 0.0001 | N.S. | N.S. |
| *D-box_cry1a_ -Luc* | p < 0.0001 | N.S. | N.S. |
| *E/D-box_per2_ -Luc* | N.S. | N.S. | p < 0.0001 |
| *E-box_per1b/2_ -Luc* | p < 0.0001 | N.S. | p < 0.0001 |

|  | **t-test** | | | |
| --- | --- | --- | --- | --- |
| **Gene** | **- CHX** | **+ CHX** | **- CHX** | **+ CHX** |
| *per2* | p < 0.05 | p < 0.01 | N.S. | N.S. |
| *cry1a* | p < 0.01 | N.S. | N.S. | N.S. |
| *tef-1* | p < 0.01 | N.S. | N.S. | N.S. |
| *e4bp4-6* | p < 0.001 | N.S. | N.S. | N.S. |
| *6-4photolyase* | p < 0.01 | N.S. | N.S. | N.S. |
| *lonrf1(2of2)* | p < 0.001 | N.S. | N.S. | N.S. |

**C**

|  | **t-test** | |
| --- | --- | --- |
| **Gene** | **LD** | **DD** |
| *tef-1* | p < 0.01 | N.S. |
| *tef-2* | p < 0.01 | p < 0.01 |
| *hlf-1* | p < 0.05 | p < 0.05 |
| *hlf-2* | p < 0.01 | p < 0.01 |
| *dbp-1* | p < 0.05 | p < 0.01 |
| *dbp-2* | p < 0.05 | p < 0.01 |
