# Supplementary material for: Regulation of per and cry Genes Reveals a Central Role for the D-Box Enhancer in Light-Dependent Gene Expression
Source: PLoS One. 2012 Dec 6;7(12):e51278. doi: 10.1371/journal.pone.0051278 (PMC3516543; doi:10.1371/journal.pone.0051278)
Supplement: Table S3 — Cosinor analysis. Period (τ) and Peak (ZT) values for all luciferase reporter constructs analyzed. “N.S.” denotes no statistically significant rhythm detected (p>0.05). (DOC) [file pone.0051278.s007.doc]

**Supplementary Table 3**

| **Construct** | **Period (τ)** | **Peak (ZT)** |
| --- | --- | --- |
| *cry1a-Luc* | 24.6$\pm$0.3 | 9.8$\pm$0.3 |
| *cry1a AP1 mut-Luc* | 24.6$\pm$0.3 | 9.5$\pm$0.1 |
| *cry1a LRR-Luc* | 23.5$\pm$0.6 | 9.6$\pm$0.2 |
| *cry1a LRR D-box mut-Luc* | N.S. | N.S. |
| *AP1-Luc* | N.S. | N.S. |
| *D-box_cry1a_-Luc* | 23.8$\pm$0.5 | 9.9$\pm$0.4 |
| *cry1a-Luc Deletion 1* | 23.7$\pm$0.4 | 9.2$\pm$0.4 |
| *cry1a-Luc Deletion 2* | 24.1$\pm$0.2 | 10.0$\pm$0.1 |
| *cry1a-Luc Deletion 3* | 24.1$\pm$0.2 | 9.7$\pm$0.4 |
| *cry1a-Luc Deletion 4* | 24.2$\pm$0.0 | 9.9$\pm$0.0 |
| *cry1a-Luc Deletion 5* | 24.2$\pm$0.0 | 10.2$\pm$0.1 |
| *cry1a-Luc Deletion 6* | 24.2$\pm$0.0 | 10.0$\pm$0.1 |
| *cry1a-Luc Deletion 7* | 24.2$\pm$0.0 | 8.9$\pm$0.0 |
| *cry1a-Luc Deletion 8* | 24.4$\pm$0.3 | 8.8$\pm$0.1 |
| *cry1a-Luc Deletion 9* | 24.0$\pm$0.2 | 9.0$\pm$0.1 |
| *cry1a-Luc Deletion 10* | 24.0$\pm$0.3 | 8.8$\pm$0.1 |
| *cry1a-Luc Deletion 11* | 23.8$\pm$0.6 | 9.0$\pm$0.5 |
| *cry1a-Luc Deletion 12* | 24.5$\pm$0.3 | 6.0$\pm$0.5 |
| *cry1a-Luc Deletion 13* | 24.4$\pm$0.2 | 7.6$\pm$0.3 |
| *cry1a-Luc Deletion 14* | 24.2$\pm$0.0 | 9.0$\pm$0.0 |
| *cry1a-Luc Deletion 15* | 24.2$\pm$0.0 | 8.7$\pm$0.0 |
| *cry1a-Luc Deletion 16* | 23.7$\pm$0.4 | 9.7$\pm$0.1 |
| *cry1a-Luc Deletion 17* | 24.1$\pm$0.2 | 8.9$\pm$0.1 |
| *cry1a LRR-Luc Sub-Deletion 1* | 23.1$\pm$0.3 | 10.0$\pm$0.1 |
| *cry1a LRR-Luc Sub-Deletion 2* | 23.3$\pm$0.0 | 10.1$\pm$0.1 |
| *cry1a LRR-Luc Sub-Deletion 3* | 23.3$\pm$0.0 | 10.1$\pm$0.1 |
| *cry1a LRR-Luc Sub-Deletion 4* | 23.1$\pm$0.3 | 9.4$\pm$0.4 |
| *cry1a LRR-Luc Sub-Deletion 5* | N.S. | N.S. |
| *cry1a LRR-Luc Sub-Deletion 6* | N.S. | N.S. |
| *cry1a LRR-Luc Sub-Deletion 7* | 22.9$\pm$0.0 | 8.1$\pm$0.2 |
| *cry1a LRR-Luc Sub-Deletion 8* | 23.6$\pm$0.3 | 9.1$\pm$0.1 |
| *cry1a LRR-Luc Sub-Deletion 9* | 23.6$\pm$0.3 | 8.6$\pm$0.2 |
| *cry1a LRR-Luc Sub-Deletion 10* | 23.6$\pm$0.3 | 9.0$\pm$0.1 |
| *cry1a LRR-Luc Sub-Deletion 11* | 23.6$\pm$0.3 | 9.9$\pm$0.1 |
| *cry1a LRR-Luc Sub-Deletion 12* | 23.1$\pm$0.3 | 9.3$\pm$0.3 |
| *cry1a LRR-Luc Sub-Deletion 13* | 23.6$\pm$0.3 | 8.5$\pm$0.1 |
