# Supplementary material for: Regulation of per and cry Genes Reveals a Central Role for the D-Box Enhancer in Light-Dependent Gene Expression
Source: PLoS One. 2012 Dec 6;7(12):e51278. doi: 10.1371/journal.pone.0051278 (PMC3516543; doi:10.1371/journal.pone.0051278)
Supplement: Table S4 — Mutagenesis primer sequences. Wild type target sequences are indicated and the mutated counterparts are highlighted in bold in the sequences. (DOC) [file pone.0051278.s008.doc]

**Supplementary Table 4**

**Mutagenesis Primers**

| **Mutagenesis Site** | **Mutagenesis Primer Sequence** | **WT Target** |
| --- | --- | --- |
| AP-1 site #1 mut. | 5’- CAAAACTTCTTGGAAAGGAATACTTGTTTGTACATAACA**TTTAGTA**ACAGTGCCATCCTGCTGCAG -3’ | 5’-TGACTCA-3’ |
| AP-1 site #2 mut. | 5’- GCTCCGTGTGCGCAATCATCAAGGT**TTTAGTA**AGTTGACCGACTGGATTGCATGGG -3’ | 5’-GGAGTCA-3’ |
| AP-1 site #3 mut. | 5’- CAGCTGTAAGTCTACGCATTGACATTTTACG**CAGACTAAGAA**TCACAACTTTCTCTACATGCGAGATATAGTA -3’ | 5’-CATAACTCAAA-3’ |
| *cry1a* D-box mut. | 5’- TATATACCGTGTTTTTCATTATTTTAAGTTATTCGGAAAAAAC**AACCTAGAGGAG**AGGGAGGCGTACTTTGCGTTGCG -3’ | 5’-AAGTTATACAAC-3’ |
