# Supplementary material for: Regulation of per and cry Genes Reveals a Central Role for the D-Box Enhancer in Light-Dependent Gene Expression
Source: PLoS One. 2012 Dec 6;7(12):e51278. doi: 10.1371/journal.pone.0051278 (PMC3516543; doi:10.1371/journal.pone.0051278)
Supplement: Table S5 — Position and size of all deletions and sub-deletions generated in the context of cry1a-Luc and cry1a LRR-Luc, respectively. (DOC) [file pone.0051278.s009.doc]

**Supplementary Table 5**

**Position and length of *cry1a* promoter deletions**

| **Deletion name** | **Deletion position and length** |
| --- | --- |
| *cry1a-Luc Deletion 1* | -1250bp to -1135bp (115bp) |
| *cry1a-Luc Deletion 2* | -1141bp to -1078bp (63bp) |
| *cry1a-Luc Deletion 3* | -1080bp to -995bp (85bp) |
| *cry1a-Luc Deletion 4* | -1006bp to -943bp (63bp) |
| *cry1a-Luc Deletion 5* | -958bp to -885bp (73bp) |
| *cry1a-Luc Deletion 6* | -891bp to -800bp (91bp) |
| *cry1a-Luc Deletion 7* | -814bp to -716bp (98bp) |
| *cry1a-Luc Deletion 8* | -749bp to -647bp (102bp) |
| *cry1a-Luc Deletion 9* | -671bp to -563bp (108bp) |
| *cry1a-Luc Deletion 10* | -584bp to -476bp (108bp) |
| *cry1a-Luc Deletion 11* | -500bp to -414bp (86bp) |
| *cry1a-Luc Deletion 12* | -421bp to -329bp (92bp) |
| *cry1a-Luc Deletion 13* | -341bp to -256bp (85bp) |
| *cry1a-Luc Deletion 14* | -274bp to -169bp (105bp) |
| *cry1a-Luc Deletion 15* | -194bp to -112bp (82bp) |
| *cry1a-Luc Deletion 16* | -129bp to -41bp (88bp) |
| *cry1a-Luc Deletion 17* | -48bp to +53bp (101bp) |
| *cry1a LRR-Luc Sub-Deletion 1* | -401bp to -387bp (14bp) |
| *cry1a LRR-Luc Sub-Deletion 2* | -394bp to -366bp (28bp) |
| *cry1a LRR-Luc Sub-Deletion 3* | -377bp to -352bp (25bp) |
| *cry1a LRR-Luc Sub-Deletion 4* | -360bp to -332bp (28bp) |
| *cry1a LRR-Luc Sub-Deletion 5* | -346bp to -323bp (23bp) |
| *cry1a LRR-Luc Sub-Deletion 6* | -335bp to -311bp (24bp) |
| *cry1a LRR-Luc Sub-Deletion 7* | -317bp to -297bp (20bp) |
| *cry1a LRR-Luc Sub-Deletion 8* | -305bp to -285bp (20bp) |
| *cry1a LRR-Luc Sub-Deletion 9* | -294bp to -265bp (29bp) |
| *cry1a LRR-Luc Sub-Deletion 10* | -281bp to -255bp (26bp) |
| *cry1a LRR-Luc Sub-Deletion 11* | -262bp to -240bp (22bp) |
| *cry1a LRR-Luc Sub-Deletion 12* | -249bp to -227bp (22bp) |
| *cry1a LRR-Luc Sub-Deletion 13* | -237bp to -215bp (22bp) |
